# Country ownership in global health

**DOI:** 10.1371/journal.pgph.0000113

**Published:** 2022-02-11

**Authors:** Abdisalan Mohamed Noor

**Affiliations:** Global Malaria Programme, World Health Organization, Geneva, Switzerland; PLOS: Public Library of Science, UNITED STATES

## Introduction

In the 1980s, the International Monetary Fund and the World Bank initiated Structural Adjustments Programmes to help developing countries whose economies were in crisis [1]. These policies were top-down, highly conditional and inflexible. They resulted in the erosion of social spending despite repeated pleas by many countries. What followed was a disaster as levels of poverty and health inequity rapidly increased in recipient countries [2]. The backlash that ensued triggered some degree of introspection by global donors. Discourse emerged on ensuring ‘country ownership’ in the design and implementation of donor supported initiatives. Two global decrees, the 2005 Paris Declaration on Aid Effectiveness and the linked 2008 Accra Agenda for Action [3] were launched to address country ownership, with donor-recipient relationships redefined as development partnerships. Since then, much of the literature in this area has focused on definitions, dimensionality and measurements [4]. Little has changed, however, in terms of the important asymmetries that underpinned the problem of country ownership in the first place.

In global health, major plans and decisions continue to be made far away from where the actual problems and solutions are, despite many hitching a ride on the ‘country ownership’ bandwagon. There is little reflection on the role of global health as much a way of rendering justice as it is of improving people’s health. Consequently, it struggles to extricate itself from the unyielding colonial legacy on which it was established, it remains unjust and at times uncaring as those who make the decisions are not those who need its succor the most. The response to the COVID-19 pandemic has been a glaring exposition of the disastrous combination of these factors [5]. In this piece, I share ten lessons I have learnt in the last 20 years on how to think about country ownership, if only to contribute to a more conscientious approach to supporting communities in need. It is my hope that all actors across global health would it find it useful, especially students and emerging leaders in this area who are likely to lead its transformation.

### 1. Country ownership is not yours to ensure, you certainly cannot confer it

If you are trying to define what ‘country ownership’ means so that your global health initiative can succeed, then you are failing already. If you are developing checklists and pathways to ‘ensure’ country ownership, then you are probably wasting your time. You are also confusing your control over resources with the value of your ideas. Instead, you must accept that countries are sovereign entities, fully responsible for planning, resourcing, implementing and monitoring their national response [6]. Your help is needed, your ideas are welcome but the solutions to country problems must be arrived at by countries themselves. Ethical partners know theirs is a supportive role.

### 2 Do not confuse government with country, or ministry of health with the health system

Often as shortcut to real engagement, many global health actors use influence over governments to push a specific agenda, thereby distorting national goals. Unfortunately, it is not unusual that many governments are not great representatives of their citizens [7]. It is also in such settings where national institutions are the weakest, most corrupt and susceptible to leverage. There is nothing as risky to the health of populations than when bad global health practice becomes bedfellow with bad governance. It is, therefore, essential that you engage across all stakeholders in society so that ideas represent the voices of citizens, especially those most in need.

### 3. Have a dialogue, ask questions, listen to those who live with the problem

Ideas that alleviate the suffering of those most in need require that you avoid preconceived notions about possible solutions to their problems. You must listen to those who are affected. As an expert, it is often tempting to be seen to provide solutions; however, avoid the urge to offer quick answers. There is a lot you don’t know, and you should spend most of your time learning instead of giving lessons. The path to good solutions is often through dialogue, including with grassroots representation, informed by the lived experiences and the decision-making culture of the country or community you are aiming to support.

### 4. ‘Evidentiary’ knowledge and control over funds create power asymmetries

By far, majority of global research funding goes to institutions and individuals in high-income countries, even for work done in low-income countries [8]. This wide funding chasm creates perverse knowledge inequity. It leads to a culture of data and information peddling [9] that is delinked from the lived experience and doesn’t speak to the rightful owners and users of this information [10], a recipe for poor policies and health outcomes. It perpetuates a chronic capacity deficit, often used by global actors to accrue even more decision-making powers. In the long run, it not only undermines true partnerships in global health but also makes mockery of the spirit of ‘great science is collaborative science’. Global research ethics and investment must address this ‘helicopter’ research crisis.

### 5. You are a helper and an ally, accept these roles and stay true to them

It takes a great amount of self-awareness to accept that, despite your lofty global position, your scientific credentials and influence over vast resources, your role is that of a helper and an ally and you are not a national leader or decision-maker. Guard against the saviour complex, be a true ally and contribute to building national capacity so that your help is needed less over time. Avoid being the center of attention, get rid of your *quid pro quo* mentality. Avoid infantilizing those who you are meant to help.

### 6. The most important voices for change are often the quietest

Global health empiricism is much the poorer for its little attempt to listen to the voices of those who need it most [11]. Women, mothers, frontline health workers, teachers, local elders, lowly government officers and other less visible members of society are the true agents of public health change. Be their champion, seek their insights and don’t underrate their ability to understand the evidence. Above all, don’t assume you know what they need.

### 7. The power of data to change minds is not simply in the ‘quality of the evidence’ but in the ‘change activism’ it catalyzes

To make data impactful, there are several well-documented systemic and technical issues related to completeness, timeliness, quality and analysis that one must get right [12]. However, the real power of data is in its ability to change minds and lives and you must, therefore, communicate it clearly and simply, tailored to the right audience. Although policy makers are important (see point 8), individuals and communities are the primary client, because they are also the most effective change agents. Empowering communities with the capacity to understand and communicate the data to its members is one of the least exploited global health resources, it could yet be its most impactful.

### 8. One size doesn’t fit all, really!

Often, global health agencies view countries, and at times regions, as homogenous entities despite the frequent rhetoric that ‘one size doesn’t fit all’. Policies and interventions that do not account for the local diversity are imposed on countries. You may have heard excuses such as: there is weak capacity to analyse data; the data is of poor quality; or the health system is not capable of delivering complex subnational programmes. However, even if available data and information are not perfect, sub-nationally tailored plans are good public health practice, engender greater sense of ownership, allow for grassroot participation, are likely to have greater impact and may eventually improve the quality of data.

### 9. Beware of the policy development addicts

As a global health expert who is continuously hopping between projects, having a favored approach included in national policy documents may bring a great deal of satisfaction for you–perhaps it might be a career highlight. Some governments, on the other hand, may accept these policies to appease its donors, even when chances of their sustainable implementation are low. Any real discussion on implementation feasibility is drowned by the voices of the ‘ambition evangelists’ who are only too happy to set lofty goals without much focus on feasibility. Beware of this self-sustaining delusion and understand that many great outcomes in life are a collection of many small wins. Being pragmatic doesn’t make you any less aspirational.

### 10. Harvesting of national data is the silent scandal of global health

In global health, there is a self-serving conflation of data sharing as a good global health practice with data extraction and vacuuming [9]. For example, timely genomic data sharing during the COVID-19 pandemic has had a huge impact on the global response, including the rapid development of vaccines [13]. However, using such examples to push countries to submit data on hundreds of health indicators, when global actors have overseen, for decades, painfully slow improvements in surveillance systems is a travesty. This cannot be absolved by endless discussions on data governance [14] that appear designed to present global health agencies with convenient tools to justify continued data extraction. Auditing donor investments [9] must not supersede meaningful strengthening of national health data and information systems. An aggressive global agenda to invest and improve national health data is urgent. The better the data the easier its sharing.

## Conclusion

If you consider global health as something you do to help those ‘vulnerable others’, then you might find the lessons in this piece burdensome. The fact is global health is not charity, and you are its first client, the abiding lesson from the COVID-19 pandemic. Understanding this will ensure you practice global health with the most powerful tool you have–empathy. If your approach to supporting countries and communities in need is one that you would find unacceptable if circumstances were reversed, stop and recalibrate. Donor investment in global health, unfortunately, is often driven by geopolitics, as powerful countries and entities try to construct or retain spheres of influence. At times, the geopolitical objectives may conflict with your conscience. Speak up when you can, importantly however, do your best to behave conscientiously, as it is the collective, sustained behaviors of individuals that shape institutional culture.
